# Anion Gap Toxicity in Alloxan Induced Type 2 Diabetic Rats Treated with Antidiabetic Noncytotoxic Bioactive Compounds of Ethanolic Extract of *Moringa oleifera*

**DOI:** 10.1155/2014/406242

**Published:** 2014-12-08

**Authors:** Maxwell Omabe, Chibueze Nwudele, Kenneth Nwobini Omabe, Albert Egwu Okorocha

**Affiliations:** ^1^Molecular Cancer Biology Research Group, Molecular Pathology and Immunology Division, Department of Medical Laboratory Sciences, School of Biomedical Science, Faculty of Health Science, Ebonyi State University, Nigeria; ^2^Cancer Research Unit, Saskatchewan Cancer Agency, Room A 231, Health Science Building, University of Saskatchewan, Saskatoon, SK, Canada S7N 5R5; ^3^Department of Pathology and Molecular Medicine and Department of Cell Physiology and Pharmacology, University of Leicester, UK

## Abstract

*Moringa oleifera* (MO) is used for a number of therapeutic purposes. This raises the question of safety and possible toxicity. The objective of the study was to ascertain the safety and possible metabolic toxicity in comparison with metformin, a known drug associated with acidosis. Animals confirmed with diabetes were grouped into 2 groups. The control group only received oral dose of PBS while the test group was treated with ethanolic extract of MO orally twice daily for 5-6 days. Data showed that the extract significantly lowered glucose level to normal values and did not cause any significant cytotoxicity compared to the control group (*P* = 0.0698); there was no gain in weight between the MO treated and the control groups (*P* > 0.8115). However, data showed that treatment with an ethanolic extract of MO caused a decrease in bicarbonate (*P* < 0.0001), and more than twofold increase in anion gap (*P* < 0.0001); metformin treatment also decreased bicarbonate (*P* < 0.0001) and resulted in a threefold increase in anion gap (*P* < 0.0001). Conclusively, these data show that while MO appears to have antidiabetic and noncytotoxic properties, it is associated with statistically significant anion gap acidosis in alloxan induced type 2 diabetic rats.

## 1. Introduction

Type 2 diabetes is a disorder of metabolism which usually affects insulin secretion and insulin uptake, with an increase in gluconeogenesis [1–3]. Type 2 diabetes is by far the most common type of diabetes accounting for 85 to 95% of cases in developed nations and an even higher percentage in developing nations [4]. Obesity, insulin resistance, beta cell malfunction, and beta cell loss are the prominent features of T2DM. Although diabetes is primarily managed by lifestyle changes and dietary modifications, administration of a pharmacological agent is required especially when treatment goals are not met [5, 6]. These conventional treatment agents include but are not limited to biguanides, sulfonylureas, thiazolidinediones, meglitinides, alpha-glucosidase inhibitors, and insulin along with a recently developed amylin analogue pramlintide [7]. Current guidelines recommend biguanide metformin as a first-line treatment, with subsequent addition of other agents when this monotherapy becomes less effective [8]. However, despite intensive therapy, glycaemic control is always difficult, leading to an increase in HbA1c levels [8].

Natural products, such as phytochemicals from plants and biologically active peptides from vertebrate and invertebrate species, are being examined for their insulinotropic properties [9]. Currently, there is a growing interest in evaluating herbal remedies, which are seen to be less toxic and have negligible side effects [10].* Moringa oleifera* is indigenous to Eastern Nigeria and is widely used in alternative medicine for treatment of cardiac and circulation problems [3, 10].* Moringa oleifera* (MO) leaves are of medicinal value and industrial uses [11]. The tree originated from Onicha in Ohaozara and across Ebonyi State in southeastern Nigeria. It is also called Ekwo/Ogbunweyo by Izzi community, in Abakaliki Local Government of Ebonyi State, southeastern geopolitical zone of Nigeria, West Africa. Also, a number of medicinal properties have been ascribed to the various parts of the plant, the root, the bark, the gum, leaf, fruit (pods), flowers, and seeds used for various ailments in the indigenous medicine of South Asia and Africa [12].

Despite the popular use of this herbal preparation for the treatment of various disorders, there is limited or no scientific data available regarding safety aspects of this remedy, nor are there any documented toxicological studies which can be used to ascertain the safety index of the herbal preparation. We and others have previously shown that aqueous extract of* Moringa* had hypoglycemic effect in rat model [3, 13, 14]. Therefore, the aim of the present study was to evaluate the antidiabetic and toxicity potential of an ethanolic extract of MO in alloxan induced diabetic rats with a view to providing information about active phytoconstituents for the clinical treatment of diabetes and its analytical toxicity index.

## 2. Material and Method

### 2.1. Plant Material

Fresh mature leaves of* Moringa oleifera* (MO) were collected from cultivation field located in Ebonyi state, Nigeria. The leaves were kept cold and protected from light during transportation and extraction processes. The leaves were dried under shade and then blended manually into smaller sizes. The pulverized leaves of* Moringa oleifera* weighing 120 g were soaked and preparedly mixed in 500 mL of 99% ethanol and allowed to stand with the bowel covered for 48 hours (2 days).

Five kilograms of MO leaves were extracted according to protocol described by Verma et al. [15]. The leaves were processed through cold solvent extraction by homogenizing with 25 L of acidified aqueous-methanol solution containing 1% acetic acid and 50% methanol. The extract was then filtered to remove the residue and concentrated by evaporating at 40°C.

The pH of the aqueous part was then adjusted to 3.5. Extract and fractions were subsequently air-dried by evaporation at ambient temperature. Fully dried extract and fractions were then weighed and stored in airtight containers at −20°C prior to analysis and further use.

### 2.2. Metformin Preparation

Metformin available in 500 mg tablet was got from Midtown Pharmaceutical Ltd. at Water Works Road Abakaliki, Ebonyi State. 500 mg of the drug was used to prepare 5 mL of metformin working solution.

### 2.3. Experimental Animal

All the animal handling and treatment protocols were done in adherence to the national and institutional guidelines for use of animals in scientific studies in Nigeria. Indeed, all the experiments were conducted in accordance with the Animal (Scientific Procedures) Act 1986 which are in strict compliance with the UKCCCR guidelines (2010) for the welfare of animals in experimental studies [16]. Each of the experiments involved at least 15 animals and was repeated at least 3 times. Adults albino rats of both sexes and the same age group (8–12 weeks) weighing 130–200 g were obtained from the Pharmacological Laboratory of the University of Nigeria, Nsukka. The animals were housed at the animal house belonging to Biochemistry Department of Ebonyi State University, Abakaliki. The rats were acclimatized for 2 weeks and fed with water and commercial livestock feed (grower). The regulatory standard for ethnical use of animals in scientific research in Nigeria was strictly observed.

**Experiment 1 (treatment with alloxan to induce type 2 diabetes).**

A single dose 84 mg/kg of alloxan Monohydrate (Sigma) dissolved in sterile PBS was used for the induction of diabetes mellitus in an overnight fasted albino Wistar rats through intraperitoneal injection of freshly prepared. The basal glucose level of the animals was measured before they were induced to have type 2 diabetes. Diabetes was confirmed after 3 days of alloxan injection by determining the fasting blood glucose concentration; only animals with fasting blood glucose 11–20 mmol/L were considered diabetic used for the experiment. The diabetic animals were allowed free access to tap water and pellet diet and were maintained at room temperature in clean iron cages.

**Experiment 2 (treatment of type 2 diabetic animals with* Moringa oleifera* extract or vehicle).**

For the second experiment, minimum of 16 rats were treated with alloxan as in Experiment 1, and after they were confirmed as diabetic; they were used for Experiment 2. The alloxan induced diabetic rats were grouped into three groups containing at least 5 rats in each group: the* Moringa* test group, metformin group, and the control group. The test group was treated with 200 mg/kg dosage of* Moringa oleifera* ethanolic leave extract. This means that 200 mg/kg of the ethanolic* Moringa oleifera* leave extract in PBS was given to each rat (the test group alone) for 5 days (morning and evening). The control or vehicle treated group received the same volume as was used in test or reference group, which is 1.5 mL of PBS at the same time and the same number of days as with the test group. After 5 days, the animals were fasted overnight and their blood samples were drawn through cardiac puncture for fasting blood glucose estimation. The blood samples were transferred into a fluoride oxalate container and were centrifuged at 3000 rpm for 15 minutes. The metformin group was treated with 10 mg/kg of metformin in PBS orally two times daily.

### 2.4. Collection of Blood Samples

Blood sample collections throughout the period of experiment were in accordance with the method of Jaiswal et al. 2009 [13]. From at least 2.5 of collected blood samples, bicarbonate levels and other parameters were immediately measured using blood gas analyser for confirmation and comparison, and other parts of the samples were transferred into lithium heparin and or potassium fluoride container which were appropriately labeled according to the identification label and were centrifuged at 3000 rpm for 15 minutes to separate plasma from the whole blood. Then each of the animals was euthanized by cervical dislocation. The separated plasma was stored in refrigerator till further use.

### 2.5. Determination of Blood Glucose Level

Glucose oxidase (GOD) catalyzes the oxidation of glucose to give hydrogen peroxide (H_2_O_2_) and glucuronic acid in the presence of the enzyme peroxidase (POD), the hydrogen peroxide is broken down, and the oxygen released reacts with 4-aminophenazone and phenol to give a pink colour. Three test tubes labeled blank, standard, and test, respectively, were set up. To all the test tubes, 1000 ul of the working reagent was added to each tube. 10 ul of standard glucose was added to the test tube labeled standard while 10 ul of sample (plasma) was added to the test tubes labeled test. The samples were mixed and incubated for 10 minutes at 37°C after which the absorbance of the standard and tests were measured against reagent blank.

### 2.6. Electrolytes Determination (Estimation)

Ion selective electrode method was used for potassium (K), sodium (Na), and chloride estimation by following the manufacturer's protocol. It is known that membrane potentials are caused by permeability of certain types of membranes to selected anions or cations. Such membranes are used to fabricate ion-selective electrodes (ISEs) that selectively interact with a single ionic species. The potential produced at the membrane-sample solution interface is proportional to the logarithm of the ionic activity or concentration of the ion in question. That was the basis on which potassium (K), sodium (Na), and chloride (Cl^−^) were measured. Bicarbonate concentration was determined by titration method.

### 2.7. Estimation of Plasma Bicarbonate Using Blood Gas Analyser and Titration Method

Immediately following blood collection, the bicarbonate level was determined using blood gas analyser. In addition titration method was also done on the same sample to ensure maximum accuracy. The principle of titration method is based on the mixing of plasma with 0.01 N hydrochloride acid, and this invariably causes a loss of acidity due to the bicarbonate in the plasma. The decrease in acidity can be determined by titrating against standard 0.01 N sodium hydroxide. To a test tube containing 1 mL of 0.01 N of HCl, 2 mL of 1% normal saline was added; 1 drop of phenol red and 0.1 mL of the plasma were added and mixed very well. Then 1 mL of 0.01 N NaOH was titrated until the last drop gave a permanent change in color

### 2.8. Determination of Lactate Dehydrogenase Level (LDH)

LDH catalyzes the oxidation of lactate to pyruvate in the presence of NAD, which is subsequently reduced to NADH. The rate of NADH formation measured at 340 nm is directly proportional to serum LDH activity. A commercially prepared kit from Sigma UK was used following the manufacturers instruction. This involved addition of 1000 *μ*L of the reagent into a test tube and then 100 *μ*L of the sample into the same tube using automatic pipette and mixed gently. The optical density was determined at 340 nm wavelength.

### 2.9. Statistical Analysis

Results are expressed as mean ± standard error of mean, and values were compared using prism statistical software (GraphPad Software Inc., UK) and Student's *t*-test. Groups of data were considered to be significantly different if *P* < 0.05.

## 3. Results

**Figure 1:**
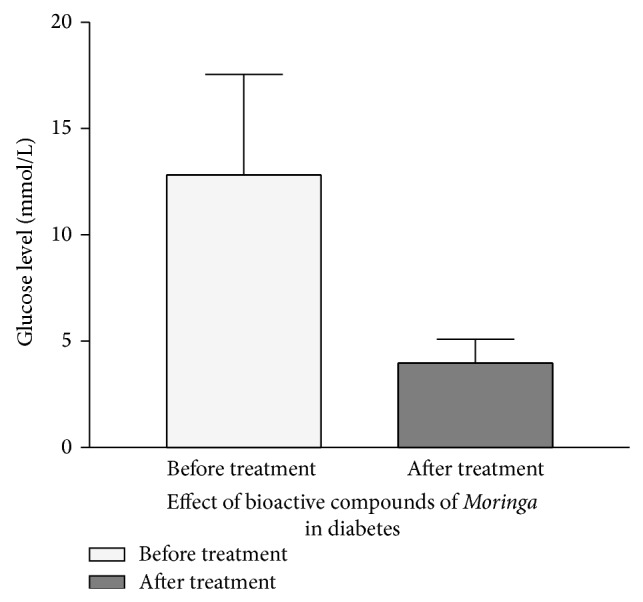
Bioactive extracts of* Moringa* reverted glucose level to normal in alloxan-induced diabetic rats. Healthy rats were confirmed as diabetic after treatment with alloxan. The diabetic rats were treated with* Moringa* extract as described in Section 2. Data show that treatment with the extract drastically reduced the glucose level. The figure is a mean and SE of 3 independent experiments.

Figure 1 compares the blood glucose level between the treated alloxan induced diabetic rats and nontreated diabetic rats. It was observed that the blood glucose level of treated diabetic rats significantly reduced by 3-fold (*P* < 0.0001) after 5 days of treatment with the ethanolic extract of* Moringa oleifera* leave compared with the blood glucose level of the nontreated diabetic group. Statistically, the mean (*x*) and standard error of mean of fasting glucose concentration of the alloxan induced diabetic rats in the treated group was 3.97 ± 1.12 mmol/L, and the mean and standard error of mean (SEM) of the blood fasting glucose concentration of alloxan induced diabetic rat in nontreated group (control group) is 13.00 ± 3.32 mmol/L.

### 3.1. Effect of Treatment on Plasma Bicarbonate

Strikingly, there was a statistically significant decrease (*P* < 0.0001) in bicarbonate levels in* Moringa* treatment and metformin treatment groups when compared with the control in Figure 4. It was also observed that metformin treatment, however, resulted in lower bicarbonate level when compared with* Moringa* treatment group. Clearly, the mean value for the control,* Moringa* treatment, and metformin treatment groups, 23.2 ± 0.4, 14.3 ± 1.3, 10.33 ± 3.1, respectively, confirmed it. Since the bicarbonate values from the control group represent a normal value for these animals, as well as in humans in clinical evaluations, it is therefore deducible that* Moringa* extract treatment resulted in mild metabolic acidosis, while metformin induced more severe metabolic acidosis.

### 3.2. Effect of Treatment on Anion Gap

Treatment with* Moringa* extract and metformin created anion gaps of 28.12 ± 1.3 and 31.4 ± 1.7, respectively, Figure 5. The anion gap value in the control group was 11.4 ± 0.4, indicating a statistically significant decrease (*P* < 0.0001) for both* Moringa* extract and reference drug metformin. The data showed that treatment with* Moringa* extract resulted in 2.8-fold increase from normal values of anion gap when compared with metformin which resulted in 3-fold increase in anion gap.

### 3.3. MO Did Not Cause a Gain in Body Weight

To determine if treatment with the extract was associated with gain or loss of weight, the weight of the animals was measured daily. Statistical analysis of the result obtained was done.  Figure 3 shows the weights of the rats. It compares the weight of the* Moringa* treated rats which have a mean value of 132.2 and standard error of ±5.05 to that of the control which has a mean value of 134.1 and standard error of 5.08. Data showed that there were no statistically significant difference in the weight of the rats (*P* = 0.8115), Figure 3. This means that* Moringa oleifera* does not cause increase in body weight, which is a known adverse effect with treatment with metformin.

### 3.4. The Lactate Dehydrogenase Level

**Figure 2:**
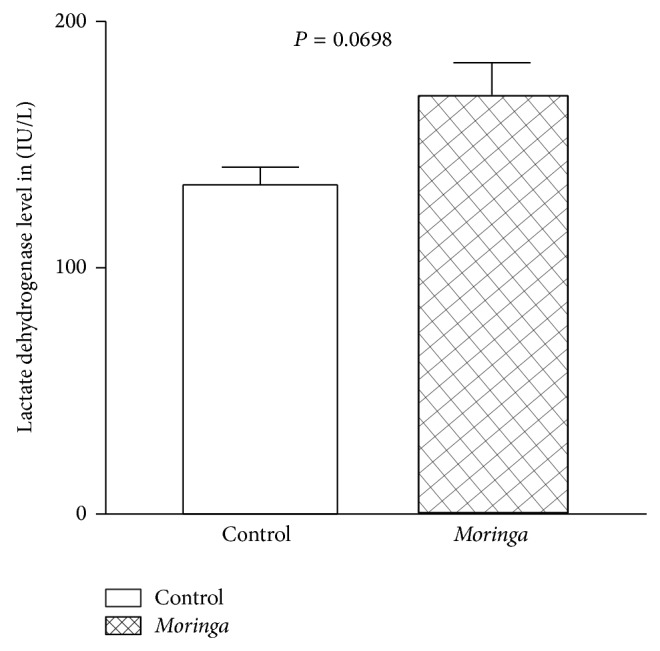
A comparison between the LDH levels in the rat when treated with ethanolic extract of* Moringa* or vehicle in alloxan induced diabetic rats. This suggests that the ethanolic extract of MO did not cause a statistically significant cytotoxicity in the animal; cleanly, there was no statistically significant difference in their LDH levels. Data is mean and SE of three independent experiments.

To determine cytotoxicity in extracted treated or vehicle treated rats, LDH concentrations in the blood samples were measured using colorimetric assay kit (Promega, Madison, WI, USA) according to the manufacturer's protocol.  Figure 2 shows the serum LDH levels of the tests which have a mean value of 170.7 U/L and standard error of 13.02 and the controls which have a mean of 133.8 U/L and standard error of 7.17. Student's *t*-test of these values at *P* = 0.0698 shows that there was no statistically significant cytotoxicity between* Moringa* treated and vehicle treated control group.

## 4. Discussion

Glucose is a major fuel for animal cells. It is supplied to the organism through dietary carbohydrates and, endogenously, through hepatic gluconeogenesis and glycogenolysis [17]. Glucose absorption from the gastrointestinal tract (GIT) into blood is regulated by a variety of neuronal signals and enterohormones (incretins), as well as by meal composition and the intestinal flora [18]. Glucose homeostasis reflects a balance between glucose supply and its utilization. Physiologically, this balance is determined by the level of circulating insulin and tissue responsiveness to it. Insulin is secreted by pancreatic islet *β* cells [18]. It stimulates glucose uptake and utilization by tissues, especially by liver, skeletal muscle, and adipose tissue [17, 18]. Medicinal plants attract growing interest in the therapeutic management of diabetes mellitus.* Moringa oleifera* is a remarkably nutritious vegetable with several antioxidant properties. The present study assessed the possible antioxidant and antidiabetic effects of an aqueous extract of* M. oleifera* leaves in treating alloxan-induced diabetic albino rats. The antidiabetic and toxic effects of aqueous extract of* M. oleifera* leaves were assessed. The current data (Figure 1) confirmed our previous report and others [3, 19–21] that the active compound of MO has strong glucose lowering potential. Alloxan and streptozotocin are widely used to induce experimental diabetes in animals. The mechanism of their action in B cells of the pancreas has been intensively investigated and now is quite well understood. The cytotoxic action of both these diabetogenic agents is mediated by reactive oxygen species; however, the source of their generation is different in the case of alloxan and streptozotocin. Alloxan and the product of its reduction, dialuric acid, establish a redox cycle with the formation of superoxide radicals, which undergo dismutation to hydrogen peroxide [22]. Thereafter, highly reactive hydroxyl radicals are formed by the Fenton reaction [22]. The action of reactive oxygen species with a simultaneous massive increase in cytosolic calcium concentration causes rapid destruction of B cells, resulting in type 2 diabetes [22]. We have shown that treatment with the ethanolic extract of MO was able to stimulate the remaining beta cells to release insulin that reduced glucose level to normal value (Figure 1).

LDH is most often measured to check for tissue damage. The protein LDH is in many body tissues, especially the heart, liver, kidney, muscles, brain, blood cells, and lungs [23]. Furthermore, we have shown lack of gain in weight and lack of statistically significant difference in cytotoxicity in rats that were treated with MO and those treated with vehicle. Lactate dehydrogenase release from damaged cells was determined and calculated. Data showed that treatment with MO was not associated with statistically significant cytotoxicity, though there was a mild increase in the LDH levels (Figure 2). However, it was observed that the ethanolic extract was rather cellular protective as the group treated with the extract had lower LDH than the control. Our data (Figure 2) is in line with the report of Asare et al. [24] and Asare et al. [25], who also demonstrated lack of cytotoxicity in rat treated with MO. In fact sufficient published works have shown that aqueous extract of MO possess strong antioxidative properties [26]. As in the current study, our data confirmed the report of Nandave et al. [26] which indicated that chronic* M. oleifera* treatment resulted in significant favorable modulation of the biochemical enzymes (superoxide dismutase, catalyses, glutathione peroxidase, lactate dehydrogenase, and creatine kinase, MB). Although we did not observe a reduced level of LDH to suggest modulation of biochemical enzymes; however, our data indicated a mild but insignificant increase in LDH in the MO treated group. Based on the results of the present study, it can be concluded that* M. oleifera* extract did not cause significant tissue damage.

**Figure 3:**
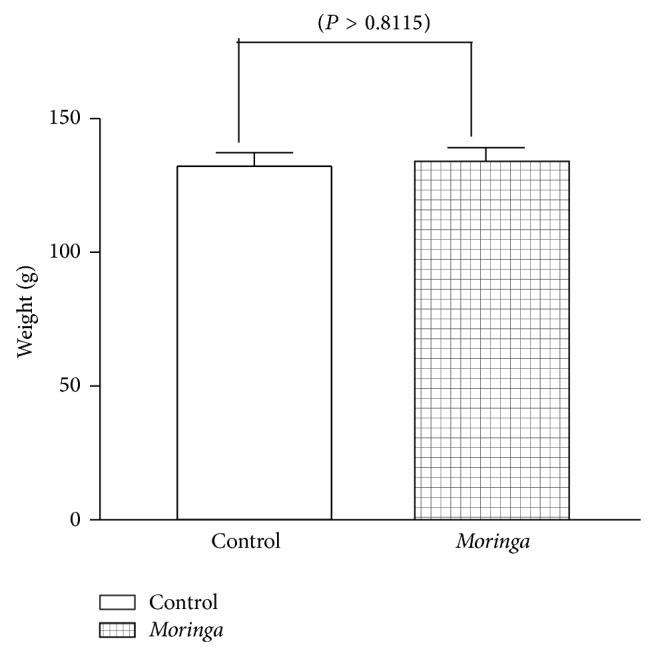
A comparison of the weights of the* Moringa* treated or vehicle treated diabetic rats; this suggests that there was no statistically significant difference in the weights of the rats. Data is mean and SE of three independent experiments.

**Figure 4:**
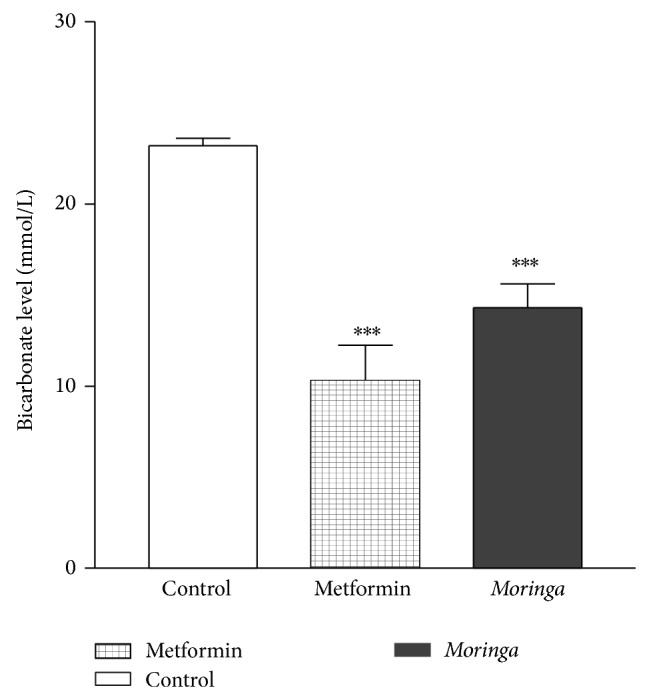
Treatment of rat with* Moringa* extract resulted in mild to moderate decreased level of bicarbonate; however, when the rats were treated with metformin, a severe decrease in bicarbonate level resulted in comparison with the vehicle treated animals. Data is mean and SE of three independent experiments ^***^ *P* < 0.0001.

**Figure 5:**
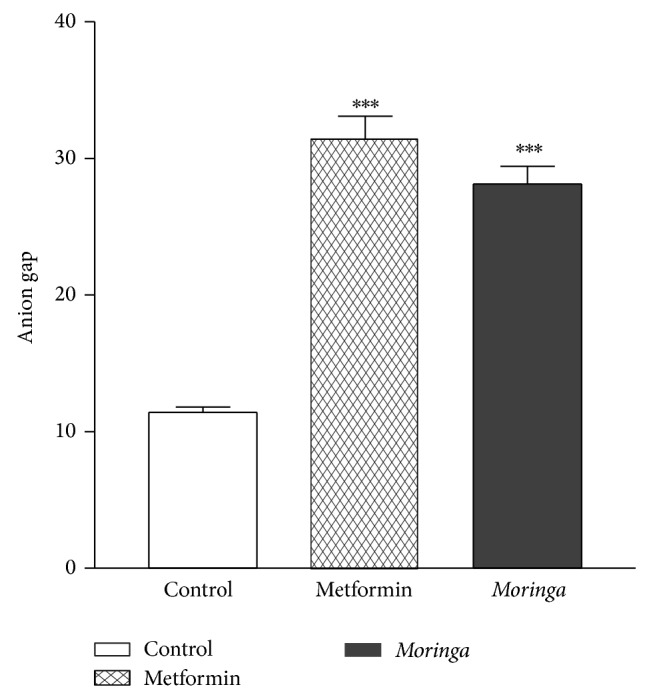
Treatment with* Moringa* extract resulted in more than 2-fold increase in the anion gap and for Metformin group there was 3-fold increase in anion gap. Thus both* Moringa* and Metformin induced anion gap acidosis compared with the vehicle treated group. Data is mean and SE of three independent experiments, ^***^ *P* < 0.0001.

Assessment of weight changes between the vehicle and extracted treated rat indicates that there were no observable gains or loss in weight in the 2 groups (Figure 3). Serum anion gap (AG) has been used to identify abnormal electrolyte concentrations [27], to detect paraproteins, and (most relevant to the nephrologists) to evaluate patients with suspected acid-base disorders [27]. The clinical approach to diagnose anion gap (AG) metabolic acidosis is to identify when it exceeds the upper limit of normal for a particular clinical laboratory measurement. The double gap metabolic acidosis provides an important clue that suggests intoxication [27]. Metformin is an oral antidiabetic biguanide that has been shown to cause metabolic acidosis [28]. To determine the safety and efficacy of MO, we investigated bicarbonate and anion gap in the diabetic rat treated with either MO or metformin. Data (Figures 4 and 5) confirmed a previous report of Bostrom et al. [28] which demonstrated that treatment with metformin was associated with anion gap acidosis. Surprisingly, our data clearly demonstrate that, like metformin, treatment with MO is also associated with metabolic acidosis and anion gap acidosis. However, it was observed that while metformin caused 2-fold decrease in bicarbonate level, MO caused 1.5-fold decrease in bicarbonate level, and both compounds are associated with strong statistically significant tripled and double anion gap toxicity in alloxan induced diabetic type 2 rats. Of note is that double anion gap metabolic acidosis provides an important clue that suggests intoxication [27]. To our knowledge, we have shown for the first time that while MO appears to have strong antidiabetic properties, its therapeutic application in type diabetes may be associated with more than a 2-fold intoxication involving anion gap acidosis, a mark of metabolic intoxication; therefore, its use may be limited to those who may not be at risk for developing acidosis. This points to the fact that anion gap would need to be monitored whenever it is used for alternative therapy for different ailments in which the herb has been shown to possess therapeutic properties. A study had reported severe acidosis with anion gap of 34 in 17 patients who were treated with metformin [29]. In the current study, anion gaps of 33 in metformin treated group and of 28 in MO treated group were observed. Clearly documented evidence indicates that severe metabolic acidosis with high anion gap intoxication had resulted in multiple organ failure and other complications especially with treatment using metformin [30].

In conclusion, these data show that while MO appears to have antidiabetic and noncytotoxic properties, its therapeutic application may be associated with statistically significant toxicity including anion gap acidosis in alloxan induced type 2 diabetic rat.
